# Urban-rural consumption differences among the elderly in developed regions: Evidence from Zhejiang, China

**DOI:** 10.1371/journal.pone.0335231

**Published:** 2025-11-26

**Authors:** Yanhong Yin, Li’ao Huang, Qiuyan Han

**Affiliations:** 1 College of International Economics and Trade, Ningbo University of Finance and Economics, Haishu, Ningbo, China; 2 Faculty of Maritime and Transportation, Ningbo University, 169 Qixinnan Road, Meishan, Beilun, Ningbo, China; University of Illinois at Urbana-Champaign, UNITED STATES OF AMERICA

## Abstract

This study examines the consumption patterns of older adults and the urban-rural disparities in Zhejiang Province, a highly developed yet rapidly aging region of China. A total of 276 valid samples were obtained from Ningbo, Hangzhou, and Wenzhou through a mixed approach combining online snowball sampling with offline random sampling. A LASSO regression model was employed to assess the effect of residence while controlling for socioeconomic variables. The results indicate that urban older adults spend significantly more than their rural counterparts, with average monthly consumption reaching 3,980 RMB compared to 2,502 RMB. Urban residence was associated with an increase of 995 RMB in total expenditure, with higher spending observed on housing, food, daily necessities, leisure and education, and health rehabilitation. Although rural respondents expressed strong interest in leisure, education, and health services, their actual expenditures in these categories were much lower, revealing a gap between intentions and behavior. Offline consumption remains dominant, but online consumption is expanding slowly. Digital exclusion persists, particularly in rural areas, due to limited literacy, complex interfaces, and unstable internet access. These findings suggest that elderly consumption is influenced not only by income but also by structural inequalities, health constraints, and digital inclusion. Policy measures should therefore strengthen rural healthcare, expand cultural and recreational opportunities, and promote elder-friendly digital platforms. Programs such as healthcare vouchers and targeted subsidies could help narrow the gap between intention and capability. This study provides preliminary exploratory insights into inclusive policies that may foster the sustainable development of China’s silver economy.

## 1. Introduction

**Fig 1:**
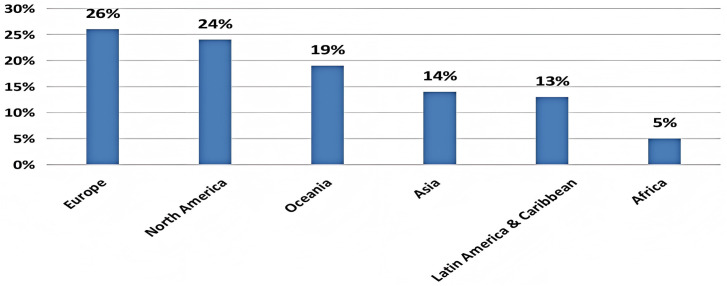
Proportion of population aged 60+ by regions.

Population aging is a global phenomenon reshaping economic and social structures. According to World Population Prospects 2024 issued by the United Nations, the elderly population (aged 60 years and above) will exceed 317 million in 2026 and 414 million in 2034 [1]. It is expected that by around 2030, more than 20% of the global population will be elderly, thus marking the transition to an “over-aged” society [1]. Although population aging has occurred primarily in developed regions or countries [1], the number of older people is steadily increasing in developing countries as well (Fig 1). As the largest developing country with the highest population, China faces the most significant aging challenge, not only in absolute numbers but also in terms of the rate of growth. By the end of 2023, the elderly population aged 60 years and above reached 297 million, accounting for 21.1% of China’s total population [2].

The elderly have emerged as a pivotal consumer group with distinct spending behaviors and needs, leading to the rise of the “silver economy,” which encompasses economic activities targeting aging populations. In China, the definition of the elderly population is undergoing gradual transformation. Traditionally, retirement ages have varied by gender and occupation: female factory workers retire at 50 years, women employed in government agencies or public institutions retire at 55 years, while men generally retire at 60 years regardless of occupation. The transition from employment to retirement often brings significant changes in individual consumption patterns and behaviors, largely due to shifts in time allocation and lifestyle. In response to these demographic and social dynamics, the scope of the silver economy in China has been conceptually broadened. As articulated in the Opinions on Developing the Silver Economy to Promote Elderly Welfare issued by the State Council, the silver economy now encompasses both the “elderly phase” (individuals aged 60 and above) and the “pre-aging phase” (individuals aged 50–60). Accordingly, this study targets residents aged 50 years and above, capturing a broad spectrum of consumption patterns and preferences within the aging demographic. This broader definition reflects a more comprehensive approach to addressing the diverse needs of an aging society and provides the foundation for more inclusive policy development and economic planning.

With the accelerating trend of population aging in China, the silver economy has become a crucial driver in addressing demographic challenges and fostering high-quality economic development. The market size of China’s silver economy reached about RMB 7 trillion (USD 970 billion) between 2023 and 2024 and is projected to expand to RMB 30 trillion (USD 4.2 trillion) by 2035, accounting for nearly 10% of the country’s GDP by that time [3]. Recognizing its importance, the Chinese government has introduced policies to enhance elderly welfare while stimulating economic growth. For example, in January 2024, the State Council of China issued “The Opinions on Developing the Silver Economy to Promote Elderly Welfare,” which proposed 26 measures to improve elderly care and support sustainable development [4]. At the national level, older adults are increasingly viewed not only as members of the labor force but also as consumers with substantial purchasing power. By 2024, the silver economy had reached a scale of RMB 7 trillion (about USD 965 billion), representing about 6% of China’s GDP [5].

Zhejiang Province, with its advanced economy and rapidly aging population, serves as an ideal setting for examining these issues. By the end of 2023, the proportion of the elderly population aged 60 years and above in Zhejiang reached 21.5%, 0.4 percentage points higher than the national average [6]. The per capita disposable income of residents in Zhejiang Province reached RMB 67,013 (about USD 9,230), the highest among all provinces in China [7]. Given the province’s advanced economic status and demographic trajectory, Zhejiang demonstrates considerable potential for the expansion of the silver economy. In this context, conducting empirical research on the consumption behaviors and preferences of the elderly population is both timely and necessary. A nuanced understanding of older adults’ consumption demands and patterns is essential for informing evidence-based policy making and the development of targeted interventions, thereby supporting the sustainable growth of the silver economy in Zhejiang.

Previous research has primarily concentrated on macro-level analyses of elderly consumption at the national scale or in less developed regions. For example, He et al. examined overall consumption upgrading among Chinese older adults [8], while Jiang explored rural elderly consumption constraints in Western China [9]. Nevertheless, detailed micro-level investigations remain limited in advanced provincial economies like Zhejiang, where fast urbanization occurs alongside marked inequalities. This absence restricts a deeper understanding of the combined effects of socioeconomic and geographic conditions on the consumption patterns and preferences of the elderly population.

Accordingly, this study seeks to address the existing research gap by systematically examining the consumption patterns, determinants, and satisfaction levels of older adults in both urban and rural areas of Zhejiang Province. The specific objectives are: (1) to analyze the distinctive consumption characteristics of urban and rural elderly populations; (2) to identify the key factors driving these consumption disparities; and (3) to evaluate the policy implications of these differences for safeguarding the rights and welfare of older adults. By providing empirical evidence on consumption heterogeneity within an economically advanced region, this study contributes to the literature on aging and consumer behavior while supporting the formulation of targeted and inclusive policies consistent with China’s National Silver Economy Plan (2024) and the UN Decade of Healthy Aging (2021–2030). Furthermore, the findings are expected to guide practical measures that promote social equity and the sustainable development of the silver economy in Zhejiang Province.

## 2. Literature review

Elderly consumption has garnered increasing attention within the context of aging and the silver economy. This review synthesizes existing literature from three perspectives: the distinctive characteristics of elderly consumption, evolving trends shaped by persistent structural divides, and overarching barriers constraining participation in consumer markets.

### 2.1. Distinctive characteristics of elderly consumption

Elderly consumption is primarily characterized by a strong emphasis on health-related and value-driven spending. Given the high prevalence of chronic illness and the growing focus on well-being, older adults allocate a substantial share of their expenditures to medical care, pharmaceuticals, and wellness products [10–13]. At the same time, value consciousness is prevalent, particularly among retirees dependent on fixed incomes [14,15]. Such consumers tend to favor high-quality, functional goods, including ergonomic aids and user-friendly appliances, which are specifically designed to address age-related physical and cognitive needs. This reflects both practical necessity and financial prudence [16].

Another defining feature of elderly consumption behavior is its strong linkage to family dynamics. Inter-generational financial transfers are widespread, often directed toward grandchildren’s education or investments in family property. In rural areas, these obligations frequently create a “sandwich burden,” compelling older adults to reduce personal wellness spending in order to meet familial responsibilities [14]. In contrast, urban elderly with income derived from pensions or real estate are generally better positioned to balance family support with personal consumption needs [17,18].

Overall, the literature suggests that elderly consumption is largely driven by health considerations and value consciousness, while remaining deeply embedded in inter-generational financial responsibilities. However, studies diverge in their assessment of the extent to which family obligations constrain personal consumption, underscoring the need for more nuanced research into how demographic and socioeconomic contexts mediate these trade-offs..

### 2.2. Evolving trends and the urban-rural divide of elderly consumption

Influenced by socioeconomic development and technological progress, elderly consumption patterns are changing. One important trend is the increasing demand for service-oriented consumption, including leisure, cultural, and wellness-related activities aimed at enhancing overall well-being [19]. In parallel, digital participation is growing. Urban older adults who are more digitally literate are increasingly using e-commerce platforms to purchase healthcare products and daily necessities [20]. These changes have further widened the urban-rural consumption gap, which is rooted in long-standing disparities in income, infrastructure, and access to services. Urban elderly, with higher disposable incomes and greater availability of public services, are more likely to participate in wellness tourism, lifelong learning, and online consumption. In contrast, rural elderly often depend on low-cost community activities such as group dancing and face significant barriers to digital access due to inadequate infrastructure and limited digital literacy. Consequently, emerging consumption patterns are predominantly concentrated in urban areas, reinforcing inequalities in consumption opportunities for older adults [20,21,23].

The literature clearly indicates that trends toward digitalization and service-oriented consumption are intensifying urban-rural inequalities. However, most existing studies remain limited to qualitative description or macro-level comparisons. Insufficiently explored is how these disparities are reflected across different consumption subcategories (e.g., healthcare and rehabilitation versus leisure and education), and how they influence older adults’ subjective consumption experiences and satisfaction.

### 2.3. Structural and psychological barriers of elderly consumption

Beyond evolving patterns and regional disparities, elderly consumers encounter structural barriers that restrict their participation in consumer markets. Financial insecurity remains a major challenge, particularly for those who depend on limited pensions or personal savings. This reduces their capacity to engage in higher-value areas of consumption such as healthcare, wellness, and tourism [22]. Accessibility is also a critical issue, as many products and services do not adequately address the needs of older users. Product design, retail environments, and customer support frequently lack appropriate adaptations for aging populations. These challenges are more pronounced in rural areas, where market incentives prioritize urban profitability [11], resulting in limited access to age-appropriate products and services for rural elderly consumers. In addition, psychological and cultural factors play an important role in shaping consumption behavior. Lifelong habits of frugality, together with social norms that prioritize family obligations over individual spending, contribute to a conservative consumption approach. Such tendencies often discourage older adults from allocating resources to self-care, leisure, and non-essential goods and services [24,25]. Moreover, elderly consumption is constrained by multiple barriers that do not operate in isolation but are interconnected and mutually reinforcing. While the literature has identified these barriers individually, few studies have analyzed how they interact within a unified framework.

## 3. Methods and materials

### 3.1. Study area

The study was conducted in both urban and rural areas of Zhejiang Province, focusing on major cities such as Ningbo, Hangzhou, and Wenzhou. Ningbo, as an economic and logistics hub, was selected for analyzing high-income consumption behaviors. Hangzhou, with advanced digital infrastructure, was well-suited for studying elderly online shopping. Wenzhou, characterized by a strong commercial environment and a higher aging population, provided insights into urban-rural consumption disparities.

### 3.2. Survey conduction

A structured questionnaire was designed to investigate consumption preferences, behaviors, and expectations among the elderly population in Zhejiang Province. The questionnaire comprised three main sections to comprehensively address the study’s objectives. The first section collected demographic and socioeconomic characteristics, including gender, age, educational attainment, occupation, income, employment status, and living arrangements. The second section examined consumption characteristics, covering consumption priorities, spending categories, expenditure patterns, and satisfaction levels across various goods and services. The third section explored personal consumption expectations, including individual challenges, aspirations, and desired improvements in the consumption environment.

All measurement scales were treated as ordinal-level variables to accurately capture respondents’ relative preferences and satisfaction levels. Specifically, Item Q12 (Attention to Consumer Information) and Item Q15 (Factors Influencing Consumption) employed a matrix-format rank-order scale, where respondents ranked seven items based on perceived attention or influence, from 1 (least important) to 7 (most important). These numerical values reflected only ordinal positioning without implying interval-level interpretation. In contrast, Item Q21 (Consumer Satisfaction) utilized a traditional 7-point Likert-type scale, whereby participants evaluated nine distinct online and offline consumption scenarios on a continuum from 1 (extremely dissatisfied) to 7 (extremely satisfied). This scale assessed the intensity of consumer satisfaction across different contexts.

Two survey methods were applied. The first was the snowball sampling technique, primarily used for online surveys. University students interviewed their grandparents, who then recommended the survey to elderly friends. The survey link, shared via a QR code generated on the online platform Wenjuanxing, was used for online responses. The second method was random sampling for offline face-to-face interactions, involving interviews with elderly individuals at community centers, markets, and other public venues.

This study was approved by the Ethics Committee of Ningbo University (No. 2024–248). Written informed consent was obtained from all participants, with strict measures taken to ensure anonymity. Data collection was carried out between March and August 2024.

### 3.3. Samples

More than 300 individuals were surveyed, and 276 valid responses were obtained, including 188 from online surveys and 88 from offline surveys. The sample covered diverse age groups, income levels, and living environments. Internal consistency reliability was assessed with a sample size of 276. The resulting Cronbach’s alpha was 0.622 (95% CI [0.547, 0.691]). According to classical reliability benchmarks [26,27], an alpha coefficient between 0.60 and 0.70 is considered acceptable. Therefore, the internal consistency of the scale meets the threshold for preliminary research, supporting its application in subsequent analyses.

Respondent demographics indicated a balanced gender distribution, with 45.7% male and 54.3% female participants. Urban residents accounted for 62.3% of the sample, while rural residents constituted 37.3%. The age distribution ranged from 50 to over 80 years, with the largest group (31.2%) falling within the 61–65 age bracket. Educational backgrounds varied considerably, ranging from illiteracy to postgraduate qualifications, with the majority having middle school or lower educational attainment.

Occupational status revealed diverse roles within the aging demographic. The largest group (52.2%) reported assisting their children as their primary activity, followed by those in active employment (27.5%). Fully retired individuals accounted for 20.3%, reflecting heterogeneity in lifestyle and economic engagement among elderly respondents.

Living arrangements showed a predominance of family-based living, with 51.4% residing with their children and 38.4% living with their spouses. Only 10.2% of respondents lived alone, indicating strong family ties and inter-generational dynamics in the region. Income distribution also varied widely: more than half (53.6%) reported annual incomes between RMB 25,000 and RMB 60,000 (monthly RMB 2,000–5,000). A significant proportion (21%) reported incomes below RMB 24,000, while an equivalent share earned between RMB 61,000 and RMB 120,000. Only a small fraction (4.35%) reported annual incomes exceeding RMB 120,000.

## 4. Results

### 4.1. Attention vs. actual spending in elderly consumption

**Fig 2:**
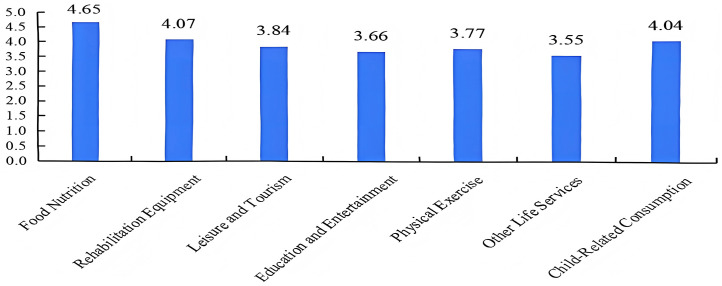
Consumption attention levels of consumption categories.

Understanding the relationship between consumption attention and actual expenditure is essential for evaluating both potential and realized consumption behaviors among the elderly. Consumption attention was assessed using a 7-point Likert scale (1 = lowest attention, 7 = highest attention), encompassing categories such as food and nutrition, rehabilitation equipment, leisure and tourism, education and entertainment, physical exercise, other life services, and child-related consumption. Descriptive statistics illustrate the attention scores of elderly individuals across these seven categories (Fig 2). “Food and nutrition” ranks highest with a score of 4.65, underscoring the priority placed on dietary health. The next highest categories are “Rehabilitation equipment” (4.07) and “Child-related consumption” (4.04), reflecting concerns with personal rehabilitation as well as family-related responsibilities. “Leisure and tourism” (3.84) and “Physical exercise” (3.77) occupy the middle range, while “Education and entertainment” (3.66) and “Other life services” (3.55) receive relatively lower scores. Overall, the findings suggest that elderly individuals primarily prioritize consumption related to health and daily living.

Despite considerable attention to leisure, education, and wellness-related activities, actual expenditure data reveal a clear discrepancy, particularly among rural elders. Table 1 presents the monthly consumption expenditure structure of elderly individuals in rural and urban areas. Food accounts for the largest share of expenditure in both groups, comprising 30.20% among rural elders and 30.56% among urban elders. Daily necessities also represent a substantial proportion, with similar shares in rural (17.70%) and urban (17.45%) populations. Medical expenses, however, are markedly higher among rural elders (16.37%) compared with urban elders (9.95%), suggesting greater healthcare needs or reduced access to adequate medical resources in rural areas. Housing expenditure is relatively higher for urban elders (9.51%) than for rural elders (7.59%), reflecting differences in living costs. While expenditures on transportation are broadly comparable, urban elders allocate a greater proportion of their spending to leisure and education (11.10%) than rural elders (8.70%), likely due to the wider availability of cultural and recreational resources in urban settings. Overall, the consumption structure demonstrates that elderly individuals primarily direct expenditures toward essentials such as food, daily necessities, and healthcare, with rural elders showing a particularly high burden of medical expenditure.

**Table 1:** Consumption expenditure structure of elders per month.

Consumption Category	Rural Elder Expenditure (RMB and %)	Urban Elder Expenditure (RMB and %)
**Housing**	159 (7.59%)	374(9.51%)
**Transportation**	169 (8.09%)	335 (8.5%)
**Food**	632 (30.20%)	1203 (30.56%)
**Daily necessities**	370 (17.70%)	687 (17.45%)
**Leisure and education**	182 (8.70%)	437 (11.10%)
**Medical service**	342 (16.37%)	392 (9.95%)
**Wellness and rehabilitation**	141 (6.37%)	273 (6.95%)
**Other expenditure**	97 (4.62%)	236 (5.99%)

### 4.2. Consumption channels and elderly satisfaction

The consumption behavior of the elderly is increasingly shaped by both traditional and digital channels. This study examined two primary modes of consumption as offline (in-person) and online (digital platform-based) by analyzing usage patterns and satisfaction levels. Survey data reveal that offline consumption continues to dominate, with the monthly expenditure ratio of offline to online spending at 0.82. However, the growing accessibility of digital technology has facilitated a gradual shift toward online consumption, particularly in categories such as leisure and entertainment (27.05%), daily necessities including clothing (21.23%), and food-related services such as fresh food delivery and takeout (14.73%). By contrast, online education (10.27%) and online ride-hailing services (5.48%) exhibit relatively low levels of engagement, reflecting varied degrees of digital adoption across different consumption domains.

**Fig 3:**
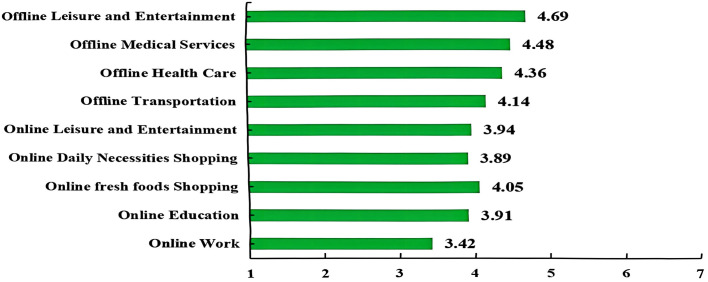
Satisfaction level of online and offline consumption.

To evaluate the quality of user experience across consumption channels, a satisfaction scale ranging from 1 (very dissatisfied) to 7 (very satisfied) was employed. The results demonstrate that offline services consistently receive higher satisfaction ratings than online services, reinforcing the preference of older adults for in-person interactions (Fig 3). For example, offline leisure and entertainment services achieved the highest satisfaction score (4.69), suggesting that social engagement and immersive experiences remain highly valued by this demographic. Similarly, offline medical and healthcare services recorded relatively high satisfaction scores (4.48 and 4.36, respectively), underscoring trust in traditional, face-to-face care models, especially in areas directly related to well-being.

In contrast, satisfaction with online services was more varied. While online fresh food purchases received a moderately positive score (4.05), online leisure and entertainment scored slightly lower (3.94), possibly due to usability challenges or limited content relevance for elderly users. Online work platforms recorded the lowest satisfaction score (3.42), highlighting a misalignment between platform functionalities and the needs or expectations of older consumers.

### 4.3. Challenges in online consumption and key improvement measures

Given the lower satisfaction levels, it is essential to examine the main sources of dissatisfaction in online consumption among the elderly, as well as their expectations for targeted improvements. The survey results indicate that usability issues are the most significant barrier, with 27.2% of respondents reporting difficulties in navigating complex interfaces and experiencing visual strain. These challenges substantially hinder older adults’ ability to engage comfortably with digital platforms. Concerns regarding product quality represent the second most frequently cited issue, with 24.28% of respondents expressing frustration over unreliable or misleading goods. In addition, 18.49% reported an inability to operate digital platforms, highlighting the persistent challenge of limited digital literacy. Other sources of dissatisfaction include restricted product variety (15.65%) and delays in logistics (14.38%), both of which reflect structural limitations in current e-commerce systems for elderly users.

In response to these challenges, elderly consumers indicated clear preferences for improvement measures prioritizing reliability, accessibility, and regulatory support. The most commonly endorsed measure was the enforcement of rigorous quality assurance standards for products intended for elderly consumers, with 18.22% highlighting the importance of ensuring product reliability and trustworthiness. An additional 16.24% advocated for the development of more age-appropriate products and the expansion of product variety, suggesting that customization and diversity are key to improving satisfaction. Regulatory interventions aimed at standardizing platform operations and safeguarding consumer rights were supported by 16.44%, revealing a strong desire for institutional protection within digital marketplaces. Government-led digital literacy programs were also viewed as essential, with 13.47% supporting initiatives to build technical skills and promote safe usage. Lastly, 12.67% expressed support for the creation of dedicated online platforms tailored specifically to elderly users, emphasizing the importance of inclusive design in fostering digital participation. In terms of enriching consumer projects, 11.49% of respondents expressed a desire for the introduction of elderly consumer education programs within communities, equivalent to the proportion advocating for increased government- or department-organized lectures on healthcare and rehabilitation products.

### 4.4. Urban-rural consumption differences among elders

The average monthly consumption expenditure of urban elderly individuals is 3,858 RMB, compared with 2,502 RMB for rural elderly individuals. Data from the Zhejiang Provincial Bureau of Statistics report a provincial per capita living consumption expenditure of 3,516 RMB, with urban residents spending 3,980 RMB and rural residents 2,539 RMB [7]. Relative to these provincial averages, the expenditure levels of the elderly population in this survey do not diverge markedly from those of the general population, suggesting that elderly individuals maintain consumption levels broadly consistent with the societal average. Nonetheless, a pronounced disparity persists between urban and rural areas. The findings indicate that the consumption expenditure of urban elderly individuals is 1.54 times higher than that of rural elderly individuals, underscoring a substantial gap in consumption patterns across the two groups.

The average monthly online expenditure of elderly individuals in urban areas is 1,269 RMB, accounting for 33.89% of their total expenditure. In contrast, their offline expenditure amounts to 2,589 RMB, representing 66.11% of their total monthly spending. For rural elderly, the average monthly online expenditure is 761 RMB, representing 30.41% of their total expenditure. Although offline spending remains the main mode for both groups, urban elderly allocate 3.48 percentage points more to online purchases than rural elderly. This gap indicates stronger engagement with e-commerce among urban elderly, which can be attributed to wider product availability and more efficient delivery services.

### 4.5. The impact of residence on elderly consumption expenditure and satisfaction

To investigate whether the type of residence (urban vs. rural) affects elderly consumption, this study employed the Least Absolute Shrinkage and Selection Operator (LASSO) method. This method is suitable for small-sample, high-dimensional data, as it addresses multicollinearity and improves model stability by shrinking less important coefficients to zero. This enables effective variable selection and supports the study’s aim of identifying key influencing factors rather than making predictions. In the model, residence type is treated as the primary independent variable, with urban areas assigned a value of 1 and rural areas assigned a value of 0. The analysis further includes several control variables, grouped into demographic attributes, economic conditions, living environment, and consumption-related factors. Demographic attributes comprise gender, age, educational attainment, and occupation before retirement. Economic conditions are measured through income level, car ownership, and possession of a driver’s license. Living conditions are captured through variables such as living arrangement and current health status. Consumption behavior is examined via factors such as information acquisition methods, the influence of different consumption drivers, and transportation choices.

The LASSO models demonstrate satisfactory performance across target variables, with R^2^ values ranging from 0.59 to 0.99, indicating strong explanatory power. Most variables exhibit low prediction errors (e.g., RMSE < 1 for satisfaction and online behavior measures), and all models converge within limited iterations, underscoring their stability and robustness.

The analysis provides strong evidence that residence is a systematic and significant predictor of both consumption expenditure and satisfaction. As shown in Table 2, urban residence is consistently associated with higher expenditure across all categories. The largest effects are observed in leisure, entertainment, and education (β = 195.04), housing (β = 189.17), and health rehabilitation (β = 105.64). For consumption satisfaction, the pattern is more complex. Urban residence increases satisfaction with online fresh food (β = 0.4415) and online work (β = 0.2305). However, it reduces satisfaction with online daily necessities (β = –0.2314) and offline healthcare (β = –0.1902). No significant effects are observed for offline transportation or medical services. Overall, the LASSO model validates that urban-rural residence is a key determinant of elderly consumption behavior, with effects differing across expenditure and satisfaction dimensions.

**Table 2:** Result of LASSO model of residence and elderly consumption.

Variable	Coefficient	Effect Description	Retained
**Total expenditure**	995.43	Urban residence → increases total expenditure by ¥995.43	Yes
**Expenditure for housing**	189.17	Urban residence → increases housing expenditure by ¥189.17	Yes
**Expenditure for food**	106.7522	Urban residence → increases food expenditure by ¥106.75	Yes
**Expenditure for daily necessities**	142.9709	Urban residence → increases daily shopping by ¥142.97	Yes
**Expenditure for leisure, entertainment, education**	195.0371	Urban residence → increases leisure/entertainment/education by ¥195.04	Yes
**Expenditure for medical**	7.074	Urban residence → increases medical expenditure by ¥7.07	Yes
**Expenditure for health** **rehabilitation**	105.6408	Urban residence → increases health rehabilitation by ¥105.64	Yes
**Other expenses**	117.9974	Urban residence → increases other expenses by ¥117.99	Yes
**Average satisfaction**	0.0038	Urban residence → increases average satisfaction by 0.0038 points	Yes
**Online work satisfaction**	0.2305	Urban residence → increases satisfaction with online work by 0.2305 points	Yes
**Online education satisfaction**	0.0356	Urban residence → increases satisfaction with online education by 0.0356 points	Yes
**Online fresh foods satisfaction**	0.4415	Urban residence → increases satisfaction with online fresh food by 0.4415 points	Yes
**Online daily necessities satisfaction**	−0.2314	Urban residence → decreases satisfaction with online daily necessities by 0.2314 points	Yes
**Online recreation entertainment satisfaction**	0.0827	Urban residence → increases satisfaction with online recreation/entertainment by 0.0827 points	Yes
**Offline transportation satisfaction**	0	Urban residence → no significant effect on satisfaction with offline transportation	No
**Offline healthcare satisfaction**	−0.1902	Urban residence → decreases satisfaction with offline health care by 0.1902 points	Yes
**Offline medical** **services satisfaction**	0	Urban residence → no significant effect on satisfaction with offline medical services	No
**Offline recreation entertainment satisfaction**	0.0961	Urban residence → increases satisfaction with offline recreation/entertainment by 0.0961 points	Yes

## 5. Discussion and policy recommendations

### 5.1. Structural roots of consumption inequality beyond income

This investigation reveals that urban elderly in Zhejiang spend significantly more than rural elderly, with overall consumption expenditure 1.54 times higher. The disparity lies not only in the total amount spent but also in the structure of expenditure. Urban elderly devote a larger share of their budgets to leisure, education, and housing, while rural elderly concentrate their spending on food and medical expenses. Health expenditure alone constitutes 16.37% of the rural elderly budget, compared with 9.95% for urban elderly. This pattern suggests that rural elderly are constrained by essential needs, which limits their capacity to engage in developmental and discretionary consumption. A similar situation has been observed in Korea, where rural elderly consume fewer nutrients such as proteins and minerals, while urban elderly benefit from a more balanced diet [28]. These disparities are deeply rooted in structural inequalities, including the uneven distribution of healthcare resources, digital access, and recreational facilities between urban and rural areas. While income redistribution programs, such as pension schemes and health insurance, are essential, they are insufficient to eliminate these structural barriers. Policies aimed at improving the consumption capacity of rural elderly must therefore address these broader systemic issues.

China has made notable progress in elderly care in recent years, particularly in urban areas. The government has expanded pension coverage and healthcare benefits, and initiated projects such as rural elderly canteens and day-care centers. Nevertheless, the quality and accessibility of these services remain uneven across regions, with rural areas still facing considerable challenges in healthcare access, social participation, and economic opportunities.

To address these gaps, public investment in cultural and recreational facilities in rural areas would enhance quality of life and create opportunities for non-essential spending. International models also offer useful insights. For instance, the “Meals on Wheels” program in the United States delivers nutritious meals and social support to elderly individuals, particularly those in isolated rural communities [29]. A similar community-based model could be adapted to rural China, where traditional family support systems are weakening and the demand for alternative social safety nets is increasing. Such a program would not only provide essential nutritional support but also foster social engagement, thereby reducing the burden on individual households and redistributing consumption patterns. This approach could be particularly beneficial in rural areas, where seniors face more severe health and social constraints. By adopting and adapting this model, China could establish a supplemental safety net that ensures access to both essential services and improved quality of life, thereby promoting a more balanced consumption structure and addressing disparities between urban and rural elderly populations.

### 5.2. The Multidimensional digital divide: Access, use, and satisfaction

Our investigation also highlights substantial gaps in digital consumption. Urban elderly allocate a larger share of their spending to online activities compared to their rural counterparts, yet both groups report dissatisfaction with digital services. The primary reasons include complex interfaces, concerns about product quality, and limited digital literacy. This indicates that the digital divide among the elderly is not solely a matter of internet access, but also of effective usage and satisfaction. In Japan, despite high internet penetration, elderly users face exclusion because digital platforms are not designed to meet their needs, and they lack adequate training [30]. Similar challenges are observed in European countries, where EU “e-Inclusion” policies emphasize improving digital skills among elderly users rather than focusing exclusively on hardware access [31].

Policy interventions should therefore aim to enhance both the accessibility and usability of digital platforms for elderly users. International initiatives provide instructive examples. For instance, “SeniorNet” in the United States offers digital education through local learning centers [32], Canada’s “Connecting Families” program provides low-income seniors with free tablets and internet services, and the United Kingdom’s “Good Things Foundation” delivers free digital training across community settings [33]. These initiatives demonstrate how tailored training and inclusive design can effectively reduce digital exclusion.

China has already acknowledged the urgency of this issue and launched initiatives such as the “Silver Digital Literacy and Skills Improvement Campaign” [34], which organizes training courses in communities and universities for seniors. Local governments have also introduced simplified versions of apps and “elder-friendly modes” on major platforms. However, these measures face persistent challenges: training coverage remains uneven between urban and rural areas, simplified apps often remain underutilized, and many rural seniors still lack stable internet access. Moving forward, policies in China should expand digital training programs, ensure that service design better reflects elderly needs, and invest in rural digital infrastructure. By integrating training, inclusive design, and infrastructure development, China can transform digital inclusion from a policy slogan into a practical driver of elderly well-being and participation in the digital economy.

### 5.3. The paradox of consumption intention and capability

The findings show that elderly individuals express strong interest in purchasing rehabilitation equipment and participating in leisure travel, yet actual expenditure in these areas remains limited, especially among rural elderly. This reflects a paradox in which high consumption intention does not translate into actual spending, primarily due to health burdens and essential costs crowding out discretionary consumption. In Korea, similar findings were observed, where rural elderly reported more health issues such as neuralgia and arthritis, leading to greater reliance on medical expenses and reduced disposable income for non-essential consumption [35].

To bridge the gap between intention and capability, targeted policy interventions are necessary. One example is the elderly healthcare voucher program launched in Hong Kong in 2009. This initiative provides seniors with medical service vouchers covering a wide range of private primary healthcare services, thereby reducing their financial burden and freeing resources for other forms of consumption [36]. In addition, China has been exploring the issuance of digital consumption vouchers specifically designed for the elderly, aimed at subsidizing spending on elderly care services and stimulating consumption in non-essential areas [37].Building on these models, China could further enhance support for the elderly by expanding access to digital platforms and offering more targeted vouchers for services such as wellness tourism, cultural activities, and recreational services. These types of direct financial support programs can help translate elderly individuals’ consumption intentions into actual spending, stimulate the development of the silver economy, and improve elderly well-being across both urban and rural regions.

## 6. Conclusion

This study examined differences in elderly consumption between urban and rural areas in Zhejiang Province, a developed coastal region in China. The analysis yielded three main findings. First, a clear gap was identified in both consumption levels and structures. Urban seniors spend about 1.54 times more than rural seniors and allocate a larger share to leisure, education, and digital services, whereas rural seniors concentrate on food and healthcare. This pattern reflects structural inequalities in access to healthcare, recreational opportunities, and social participation. Second, a paradox was observed between intentions and actual spending. Many elderly individuals, particularly in rural areas, express strong interest in rehabilitation equipment and leisure activities, yet essential expenditures and health burdens constrain their ability to realize these intentions. Third, while offline consumption continues to dominate, online consumption is gradually expanding. However, digital exclusion remains a significant challenge due to limited literacy, complex interfaces, and unstable internet access in rural communities.

This exploratory study, based on a small sample from one province, provides limited evidence but reveals important patterns in elderly consumption. It demonstrates that consumption is influenced not only by income but also by structural conditions, digital access, and health constraints. Policy measures should therefore extend beyond income redistribution to include the expansion of rural healthcare, enhancement of cultural and recreational facilities, and development of elder-friendly digital platforms. Initiatives such as healthcare vouchers and targeted subsidies could help bridge the gap between intentions and actual spending. International models, including community-based nutrition programs and digital literacy initiatives, offer valuable insights that could be adapted to the Chinese context.

Limitations and directions for future research should be acknowledged. The reliance on a small sample from one province restricts the generalization of the findings. Moreover, the cross-sectional data cannot capture temporal changes, limiting the ability to trace how elderly consumption evolves with aging and social transformation. Future studies would benefit from larger and more diverse samples, longitudinal designs, and comparative analyses across provinces or countries. Such approaches could provide more robust evidence and strengthen the policy relevance of the patterns identified here.

## Supporting information

S1 Appendix Elderly consumption survey. (PDF)
