# Supplementary material for: Urban-rural consumption differences among the elderly in developed regions: Evidence from Zhejiang, China
Source: PLoS One. 2025 Nov 26;20(11):e0335231. doi: 10.1371/journal.pone.0335231 (PMC12654945; doi:10.1371/journal.pone.0335231)
Supplement: S1 Appendix — (PDF) [file pone.0335231.s001.pdf]

Appendix 1

Elderly Consumption Survey

I. Basic Attributes [Single-choice Question]

Your gender ()

○ A. Male

○ B. Female

2. Your age () [Single choice question]

○ A. 50–55

○ B. 56–60

○ C. 61–65

○ D. 66–70

○ E. 71–75

○ F. 76–80

○ G. Over 80

3. Your educational level () [Single choice question]

○ A. Illiterate

○ B. Primary school

○ C. Middle school

○ D. High school or technical secondary school

○ E. Undergraduate or associate degree

○ F. Postgraduate

4. Your occupation before retirement () [Single choice question]

○ A. Private or civil enterprise employee

○ B. State-owned enterprise/government/public institution employee

○ C. Self-employed (business or trade)

○ D. Unemployed/housewife

○ E. Farmer

○ F. Casual laborer

5. Your current status () [Single choice question]

○ A. Still working

○ B. Retired and fully enjoying retirement

○ C. Retired, assisting children (housework or childcare)

6. Your living arrangement () [Single-choice question]

○ A. Living alone

○ B. Living with a spouse

○ C. Living with children

7. Your current annual income ( ) in ten thousand RMB [Fill in the blank]

8. Your place of residence () Province () City [Fill in the blank]

9. Your residence type () [Single-choice question]

○ A. Urban

○ B. Rural

10. Do you own a car? () [Single-choice question]

○ A. Yes

○ B. No

11. Do you have a valid driver's license? () [Single-choice question]

○ A. Yes

○ B. No

12. Which consumption information are you most concerned about? Rank from 1 to 7

(1 being the most important):

A. Food and nutrition (food, nutritional supplements, and health products)

B. Rehabilitation equipment (smart monitors, multifunctional care beds, electric wheelchairs, etc.)

C. Leisure and tourism (travel, farm stays, etc.)

D. Education and entertainment (night school, learning skills, singing, dancing, photography, etc.)

E. Physical exercise (equipment, venues)

F. Other life services (shopping, canteens, online games and entertainment, financial services)

G. Child-related consumption (daycare, interest classes, canteens)

13. How do you obtain information? (Multiple choice)

□ A. Online platforms such as WeChat

□ B. Radio

□ C. Television

□ D. Phone calls with friends or relatives

□ E. Email

14. How often do you check information? () [Single-choice question]

○ A. Every few hours

○ B. Once a day

○ C. Every 2–3 days

○ D. Once a week

○ E. Once a month

○ F. Every few months

15. Your monthly online consumption expenditure () RMB [Fill in the blank]

16. Your monthly offline consumption expenditure () RMB [Fill in the blank]

17. Your detailed monthly consumption expenditure (approximate amounts):

Housing loans and rent () RMB [Fill in the blank]

Transportation (car loans, insurance, fuel, public transport) () RMB [Fill in the blank]

Food and dining (groceries, tobacco, alcohol, takeout) () RMB [Fill in the blank]

Daily shopping (clothing, household goods) () RMB [Fill in the blank]

Leisure, entertainment, and education () RMB [Fill in the blank]

Medication, hospital visits, and healthcare () RMB [Fill in the blank]

Wellness and rehabilitation () RMB [Fill in the blank]

Other expenditures () RMB [Fill in the blank]

18. What is your primary mode of transportation? (Multiple choice)

□ Walking

□ Bicycle, electric bike, motorcycle

□ Subway

□ Buses

□ Taxi or ride-hailing services

□ Private car

19. What types of online consumption do you engage in? (Multiple choice)

□ A. Daily shopping (clothing)

□ B. Fresh groceries and takeout

□ C. Education and learning

□ D. Ride-hailing and taxi services

□ E. Leisure and entertainment (e.g., Douyin, WeChat)

□ F. Medical and wellness products

□ G. Buying or renting properties

20. How satisfied are you with the following types of consumption? Rank from 1 to 7 (7 being the highest satisfaction):

A. Online work

B. Online education

C. Online grocery shopping

D. Online daily goods shopping

E. Online leisure and entertainment

F. Offline transportation

G. Offline wellness services

H. Offline healthcare

I. Offline leisure and entertainment

21. What are the main reasons for dissatisfaction with online consumption? (Multiple choice)

□ A. Limited options

□ B. Delayed logistics

□ C. Poor or unreliable quality

□ D. Complex operations, hard to navigate

□ E. Lack of ability to operate

22. To improve consumption satisfaction, which of the following changes do you most hope for? (Multiple choice)

□ A. Establishing online consumption platforms specifically for elderly users

□ B. Developing more elderly oriented products and increasing product variety

□ C. Conducting strict quality checks for elderly products to ensure reliability

□ D. Enacting regulations to standardize platform operations and protect elderly consumer rights

□ E. Organize government-led training to increase the skills and safety awareness of elderly consumers

□ F. Increasing the number of healthcare and rehabilitation-related lectures organized by the government or institutions

□ G. Bring elderly consumer education lectures into communities

23. Regarding consumption, what changes do you most hope for? (Multiple choice)

□ A. Changing consumption methods and training elderly users in online shopping

□ B. Changing consumption categories and increasing spending on physical exercise and healthcare

□ C. Reducing total consumption to increase savings for risk prevention

□ D. Increasing spending on leisure, entertainment, and clothing to enjoy life more

□ E. Maintaining the current consumption structure and spending more time with family

□ F. Other or no change

24. What measures do you most hope the government will take to promote elderly consumption? (Single choice)

○ A. Issue elderly consumption vouchers or living subsidies for eligible groups

○ B. Subsidizing merchants and enterprises for elderly rehabilitation products to lower prices

○ C. Provide rental subsidies for rehabilitation medical products, covering 50%-70% of rental fees

○ D. Increasing the variety of elderly specific products

○ E. Optimizing the consumption environment, especially online safety and protection

○ F. Conduct training or lectures to increase elderly consumers' safety awareness and skills

25. If the government implements the above measures, how do you estimate that your future consumption will change? (Matrix Single-choice Question)

Increase | Decrease | Remains the same

Housing or rent expenditure

Transportation expenditures (car loans, insurance, fuel, public transport)

Food and dining expenditures (groceries, tobacco, alcohol, takeout)

Daily shopping expenditures (clothing, household items)

Leisure, entertainment, and education expenditures

Healthcare and wellness expenditures

26. What types of rehabilitation and healthcare products do you primarily use? (Multiple choice)

□ Personal medical assistive devices (e.g., nebulizers, oxygen concentrators, blood pressure monitors)

□ Skill training assistive devices (e.g., training stairs)

□ Orthotics and prosthetics (e.g., orthopedic shoes, prosthetic limbs)

□ Personal self-care and protective assistive devices (e.g., raised toilet seats, shower chairs, commode chairs)

□ Personal mobility assistive devices (e.g., electric wheelchairs, manual wheelchairs, walkers, canes)

□ Household assistive devices (e.g., anti-shake utensils, adaptive dining tools)

□ Furniture and adapters for home and other spaces (e.g., movable dining tables, wall grab bars)

□ Communication and information assistive devices (e.g., hearing aids, visual aids)

□ Object handling and tool assistive devices (e.g., grabbers for the elderly)

□ Environmental improvement and assessment devices (e.g., measurement tools)

□ Employment and vocational training assistive devices (e.g., workplace furniture and decorative elements)

□ Recreational assistive devices (e.g., pedal exercisers)

27. For the following rehabilitation products, what do you estimate your consumption trends will be over the next five years? (Matrix Single-choice Question)

Increase | Decrease | Remains the same

Personal medical assistive devices (e.g., nebulizers, oxygen concentrators, blood pressure monitors)

Skill training assistive devices (e.g., training stairs)

Orthotics and prosthetics (e.g., orthopedic shoes, prosthetic limbs)

Personal self-care and protective assistive devices (e.g., raised toilet seats, shower

chairs, commode chairs)

Personal mobility assistive devices (e.g., electric wheelchairs, manual wheelchairs, walkers, canes)

Household assistive devices (e.g., anti-shake utensils, adaptive dining tools)

Furniture and adapters for home and other spaces (e.g., movable dining tables, wall grab bars)

Communication and information assistive devices (e.g., hearing aids, visual aids)

Object handling and tool assistive devices (e.g., grabbers for the elderly)

Environmental improvement and assessment devices (e.g., blood glucose measurement tools)

Employment and vocational training assistive devices (e.g., workplace furniture and decorative elements)

Recreational assistive devices (e.g., pedal exercisers)
